# Social Media Addiction and Mental Health Among University Students in Saudi Arabia: A PLS-SEM Analysis with Study Discipline as a Moderator

**DOI:** 10.3390/healthcare14131862

**Published:** 2026-06-26

**Authors:** Alaa M. S. Azazz, Ibrahim A. Elshaer

**Affiliations:** 1 Social Studies Department, College of Art, King Faisal University, Al-Ahsa 31982, Saudi Arabia; 2 Management Department, College of Business Administration, King Faisal University, Al-Ahsa 31982, Saudi Arabia

**Keywords:** social media usage, anxiety, stress, depression, university students, mental health disorder, SA

## Abstract

Background: The rapid use of social media (SM) has become a central part of university students’ everyday habits. However, its extensive SM surfing, frequently conceptualized as Social Media Addiction (SMA), is accountable for growing worries about the potential relationship with mental health symptoms (MHS). This research paper aimed to explore the interrelationships between SMA and MHS in the Saudi Arabia (SA) context. The paper explored the role of study discipline as a moderator. Methods: Partial Least Squares Structural Equation Modelling (PLS-SEM) technique was used to analyze a set of data collected from 600 university students in SA. Results: The PLS-SEM results showed that Time Management & Performance (TM&P) and Social Comfort (SC) were significantly and positively correlated with stress, anxiety, and depression symptoms among SA university students. TM&P exhibited the strongest association with distress symptoms, while SC also displayed significant positive associations with all mental health symptoms. In contrast, Withdrawal & Health Problems (W&HP) demonstrated weak, negative and significant correlations with stress and anxiety and a non-significant correlation with depression, indicating that different aspects of SMA might be associated with mental health outcomes differently. Moreover, study discipline can significantly moderate several relationships between SMA dimensions and mental health outcomes, signalling that the psychological associations of compulsive social media use vary across disciplinary settings. Conclusion: This research contributes to the growing body of knowledge on the association between digital addiction and psychological well-being and provides a culturally grounded perspective from the Saudi Arabian context.

## 1. Introduction

The integration of social media networks into university students’ everyday lives has profound effects on their mental health (MH), largely in higher education settings. Social media platforms such as “WhatsApp, Snapchat, Instagram, and TikTok” have changed the ways of communication, the education process, and self-expression for all residents in Saudi Arabia (SA). This country is recognized as having one of the highest levels of digital penetration globally [1].

Despite a growing body of evidence linking social media addiction (SMA) to mental health symptoms (MHS) [2,3,4,5,6,7,8,9], numerous research gaps remain, particularly in the SA context. To the best of the author’s knowledge, this paper is the first attempt to explore the relationships between SMA, as operationalized with the excessive social media use dimensions adapted from Hou et al. (2014) [3], with MHS employing the “Depression, Anxiety, and Stress Scale” (DASS-21) in SA settings with study discipline as a moderator variable. Previous studies have shown a positive correlation between SMA and some psychosocial factors like depression, anxiety, and stress among several populations. For example, extreme SM usage was found to be related to higher levels of stress, anxiety, and depression [2]. Similarly, a previous study using medical and dental university students as the study sample frame found a positive association between SMA and MHS, highlighting the role of emotional regulation [3]. However, previous evidence did not examine potential moderators (i.e., study discipline) that might affect the relationships between SMA and MH outcomes. The current study sought to further explore this gap by using PLS-SEM to test the associations between SMA and MHS, while accounting for the core moderating role of study discipline. The research aims to go beyond simple associations to develop a thorough framework that integrates both direct, indirect and moderating mechanisms. Specifically, it assesses whether the relationship between SMA and MHS, such as anxiety, depression, and stress, is structured by moderators like study discipline. By doing so, the study aims not only to identify how digital behaviors affect student MH but also to evaluate whether particular groups of university students are disproportionately at risk.

## 2. Literature Review and Hypotheses Justification

The present study is supported by the “Compensatory Internet Use Theory (CIUT)” proposed by Kardefelt-Winther [4], which argues that people may be excessively engaged in online behaviours as a coping strategy to compensate for adverse emotions, stress, loneliness, or unfulfilled emotional needs. According to this theory, social media platforms might offer temporary psychological relief, social support, and distraction from real-life challenges. However, prolonged dependence on social media as a compensatory mechanism might gradually develop into maladaptive patterns of attitude and addictive behavior, which can adversely affect emotional well-being. Within the university settings, students encountering academic challenges, psychological distress, or social isolation might gradually rely on social media networks for psychological comfort and social support, possibly escalating symptoms of anxiety, stress, and depression over a long time. Therefore, CIUT offers an important theoretical background for recognizing the psychological mechanisms triggering the associations between social media addiction and mental health symptoms among SA university students.

Recent evidence suggests that the consequences of problematic social media use extend beyond psychological distress and may also affect users’ information-processing behaviors. A systematic review by La Selva et al. [5] found that problematic social media use is associated with a greater likelihood of engaging with and sharing misinformation and fake news. The authors argued that excessive engagement with social media platforms may increase impulsive online behavior, reduce critical evaluation of online content, and heighten vulnerability to misleading information.

Extreme involvement in digital platforms such as SM can cause addiction-like indicators similar to substance-related symptoms. These might include symptoms such as deep salience, high tolerance, mood variation, conflict with others, withdrawal, and health problems (W&HP) [6,7]. The withdrawal factor describes the negative feelings and physiological responses (such as irritability, impatience, or sadness) that occur when people overuse these digital technologies or are unable to access them [8]. Withdrawal symptoms are thoroughly related to elevated levels of stress. When people are regularly and intensively engaged on online platforms, several physiological symptoms and frustration reactions can be triggered, leading to acute stress [9,10]. Previous research has declared that people with a higher level of withdrawal behavior have a significantly higher level of stress disorder [11]. Likewise, depression symptoms repeatedly co-occur with extreme social media usage, and withdrawal behavior may act as a tool that intensifies depression states [12,13].

Furthermore, feelings of anxiety is intensely linked with the withdrawal element of digital technology addiction [14,15]. If, sometimes, access to social media technologies is restricted, people practice what is called “a fear of missing out” (FoMO), contributing to a heightened level of anxiety [16]. Hence, the hypotheses below are proposed:

**H1.**  *W&HP symptoms can be positively correlated with stress symptoms.*

**H2.**  *W&HP symptoms can be positively correlated with depression symptoms.*

**H3.**  *W&HP symptoms can be positively correlated with anxiety symptoms.*

One critical factor within the SMA construct is time management and performance (TM&P), which describes users’ inability to manage the volume of time spent on social media networks, leading to procrastination, task abandonment, and reduced academic performance [8]. Poor time management is repeatedly linked with extreme SM usage and has been broadly acknowledged as a key forecaster of psychological stress and degraded well-being [17]. Compulsive use of social network sites (SNS) can consume substantial time that might otherwise be devoted to productive practices, leading to feelings of guilt, burnout, and stress [18]. Previous evidence indicates that abnormal social media use can disrupt self-regulation, weakening people’s capacity to focus on their main responsibilities and handle workload efficiently [11]. This poor time management repeatedly creates a loop of avoidance behaviour and discomfort [19]. People who fail to manage their social media use frequently experience increased stress due to time pressure and task accumulation [20]. Likewise, the constant connectivity associated with social media engagement might exacerbate cognitive overload and emotional exhaustion, which are closely linked to stress warning signs [15].

Additionally, poor time management caused by extreme SM usage has been associated with depressive symptoms through several mechanisms, involving decreased productivity, academic collapse, and adverse self-comparison [19]. When people perceive that their online surfing interferes with actual goals, feelings of low self-worth and regret may develop steadily, strengthening the depression symptoms [21]. Furthermore, excessive time spent on social media surfing often exposes people to idealized views of others’ activities, which can reinforce the feeling of depressive cognition [18]. Problem in managing time due to SM misuse is also linked with an expanded feeling of anxiety. The need to stay constantly linked, combined with the “fear of missing out” (FoMO), creates anticipatory anxiety tension [16]. Previous research showed evidence that people who spend excessive time on social media networks reported higher levels of anxiety symptoms due to decreased control over their digital activities and everyday routines [10]. Hence the hypotheses below can be introduced:

**H4.**  *TM&P can be positively correlated with stress symptoms.*

**H5.**  *TM&P can be positively correlated with depression symptoms.*

**H6.**  *TM&P can be positively correlated with anxiety symptoms.*

Social media networks can provide a virtual universe for people to communicate, connect, and obtain social recognition, reinforcing a feeling of social comfort (SC) [8]. From the perspective of SMA, SC represents a key psychological approach through which people rely on online communication to meet unmet social and emotional needs [22]. When individuals feel loneliness and/or social anxiety, they may seek out the SM space as a compensatory means of communication and belonging [13]. Nonetheless, the dependence on these digital tools for interaction with others can develop into a habitual behaviour, leading to maladaptive psychological consequences [12]. People who rely heavily on social media networks (SMNs) for emotional support often report higher levels of stress. This is because the emerging need for repeated connection can foster emotional strain, particularly when participants’ expectations are not met or when negative feedback is returned [15]. The continual pressure to be online, to reply to messages immediately, and to stay active online can also create a cognitive load, thereby increasing stress levels [23].

Social comfort (SC) via SM engagement can be a precursor of depressive symptoms. People who primarily seek emotional support online frequently experience social comparison and disappointment when connections do not fulfil their expectations [18]. The distance between the perceived online reassurance and actual social isolation may maximize the feelings of loneliness and sadness, reinforcing depressive symptoms [19]. Previous studies found that SC (caused by social media engagement) can act as an avoidance coping mechanism, reinforcing depression via patterns over a long time [6]. SC (based on social media experience) is also closely linked with anxiety, especially in people with high sensitivity to social assessment. FoMO and a continual need for online support can increase social anxiety and fear of denial [16]. Elhai et al. [10] argued that people who rely on digital networks for comfort repeatedly experience intensified anxiety. Therefore, the hypotheses below can be supposed:

**H7.**  *SC can be positively correlated with stress symptoms.*

**H8.**  *SC can be positively correlated with depression symptoms.*

**H9.**  *SC can be positively correlated with anxiety symptoms.*

The associations between SMA and MHS (stress-depression-anxiety) might not be the same across all people; contextual variables such as study discipline can play a moderate role in this context. The study discipline: social science (business schools, colleges of arts, and colleges of law) and natural science (colleges of computer science, engineering, agriculture and food sciences, as well as health-related colleges) can form the academic setting, and the ways of digital media involvement [24]. Students enrolled in natural science disciplines might use social media as an extension of their academic life, whereas those in social science disciplines might use it mainly for social networking and emotional support [25,26]. These differences might influence how the various factors of SMA (time management and performance, withdrawal symptoms, and social comfort) can be associated with mental health outcomes. For example, students enrolled in disciplines that widely depend on online teamwork and digital tools, such as computer science, withdrawal from online connections may cause disruptive, worsening stress and anxiety feelings [24]. On the contrary, students in disciplines with less reliance on online resources (e.g., social sciences) may experience weaker withdrawal effects because their academic duties are less closely tied to digital media [27]. Therefore, the study discipline can increase or decrease the relationships between withdrawal symptoms and MHS. Likewise, students in demanding fields of study such as engineering and medicine, where time pressure is high, are more exposed to the adverse outcomes of poor time management caused by extreme social media engagement [28].

In contrast, students in more flexible fields of study may have less academic-related stress [28]. Furthermore, students enrolled in social science disciplines are frequently engaged in reflective, interactive learning contexts that strengthen emotional sharing, possibly finding greater comfort and reduced loneliness through social media engagement [29]. However, students in other medicine or engineering fields may perceive less emotional relief from social media usage and possibly undergo degraded protective element against stress [30].

**H10–H11–H12.**  *Study discipline can moderate the association between W&HP and MHS.*

**H13–H14–H15.**  *Study discipline can moderate the association between TM&P and MHS.*

**H16–H17–H18.**  *Study discipline can moderate the association between SC and MHS.*

## 3. Methods

### 3.1. Research Design

This study adopted a cross-sectional survey approach, employing a structured questionnaire to test the justified relationships among the study’s main constructs. Such an approach is adequate, as it facilitates the collection of data from a large number of respondents within a limited timeframe and enables the analysis of complex relationships through the PLS-SEM technique [31], employing SmartPLS v4 (GmbH, Monhein and Rhein, Germany).

### 3.2. Participants

The data collection targeted students from five SA universities, representing several geographical regions. Specifically, 25% of the respondents were from King Faisal University (KFU) in the eastern region, 24% from Umm Al-Qura University (UQU) in the western region, 23% from Imam Mohammad Ibn Saud Islamic University (IMAMU)in the central region, 15% from Jazan University (JazanU) in the southern region, and 13% from Northern Border University (NBU) in the northern region. This geographical spreading improved the representativeness of the sample by including students from several regional backgrounds across SA. A total of 800 questionnaires were distributed. The inclusion criteria required contributors to: (1) be currently enrolled in SA university, (2) actively use at least one SM platform, and (3) voluntarily contribute to the study through informed consent. The exclusion criteria included: (1) incomplete questionnaire answers, (2) responses with extreme missing data, and (3) people who were not presently enrolled as university students at the time of collecting data. Students were informed of the study’s main aim and reassured that their replies would be kept anonymous and confidential.

Following the primary screening process, 200 forms were excluded due to incomplete questionnaires, excessive missing data, or non-current student participants. Accordingly, 600 valid forms were retained for further analysis. The final sample size (600) was considered acceptable for Partial Least Squares Structural Equation Modeling (PLS-SEM), as it considerably exceeded the minimum sample size (384) recommendations commonly suggested in quantitative studies [32] or in the PLS-SEM literature [33] and offered sufficient statistical power to evaluate the suggested structural and moderation associations. Participants were from two major study disciplines consistent with prior higher education and digital behaviour literature [34,35]: social sciences and natural sciences. Students from social science disciplines (Business Schools, Colleges of Arts, and Colleges of Law) represented 52% of the sample. Meanwhile, students from natural science disciplines (Colleges of Computer Science, Engineering, Agriculture and Food Sciences, as well as Health-Related Colleges) accounted for 48% of the sample. Regarding gender distribution, males accounted for 55% of the responses, and females accounted for 45%. In terms of age, the majority of respondents (70%) were between 18 and 24 years old, followed by 25% aged 25 to 30, while only 5% were over 30.

### 3.3. Instruments and Procedures

Social media addiction was operationalised using 10 items, as suggested by Hou et al. [3]. Participants were asked to indicate the frequency of their social media usage experiences by responding to the questions on a five-point Likert scale ranging from 1 (“Never”) to 5 (“Always”). The scale has three main dimensions: W&HP (4 items), sample item includes “How often do you feel anxious, irritable, or depressed when you are unable to access social media?”, TM&P (3 items) sample items example is “How often do you neglect your daily responsibilities because of using social media?”, and SC (3 items), sample item includes “How often do you feel more comfortable interacting on social media than in real-life situations?”. Likewise, MHS was measured through DASS-21 scale with three main factors (anxiety, stress, and depression) as recommended by Lovibond [36]. DASS-21 is considered one of the most widely used measures to evaluate MHS and has been demonstrated to have good psychometric properties in previous studies [34,37,38,39,40]. Response options of DASS-21 scale ranging from 0 = “Did not apply to me at all” to 3 = “Applied to me very much or most of the time”. Participants were expected to indicate the extent to which they experienced symptoms (stress, anxiety, and depression) of mental health during a specified period (last week). The first dimension of the DASS-21 scale has 7 variables designed to measure depressive symptoms; a sample item is “I was unable to become enthusiastic about anything”. Similarly, the second factor of the DASS dimension is structured to measure anxiety symptoms, with 7 reflective questions, and the sample item is “I felt close to panic”. The third and last dimension was developed to stress symptoms, with 7 questions; a sample item is “I found it difficult to calm down”.

Furthermore, the designed questionnaire was translated employing the back-translation procedure to guarantee linguistic accuracy. The original version (English version) was translated into Arabic by a bilingual academic, and an independent scholar afterwards translated the Arabic version back into English. Both versions were precisely compared to confirm conceptual consistency and semantic equivalence before the final distribution of the questionnaire. Study data were collected through an online survey during October and November 2025, administered and developed on a digital platform (Google Forms) to ensure accessibility and wider geographic coverage. The link to the developed questionnaire was disseminated with the assistance of colleagues across several Saudi Arabian universities, who facilitated student admissions by distributing the questionnaire via institutional email, social media platforms, and student groups. This distribution mechanism increased the response rate and ensured representation across diverse regions of SA.

To minimise the potential effects of common method bias (CMB), several procedural and statistical steps were taken. Procedurally, respondents were assured of their confidentiality and anonymity to minimize social desirability bias. Furthermore, the questionnaire items were derived from well-established, previously validated measures and were thoroughly translated and back-translated to ensure conceptual clarity and minimise item ambiguity and to ensure the validity and equivalence of the two language (English and Arabic) versions of the developed scale. Statistically, Harman’s single-factor test was implemented using an exploratory factor analysis (EFA) to assess whether a single factor could explain most of the variance among the scale items. The findings demonstrated that the unrotated first factor explained only 34.5% of the total variance, which is below the commonly accepted cutoff of 50%, suggesting that CMB is not a problem in this study.

## 4. Results

Henseler et al.’s [41] recommendations of a two-tier analysis process for a PLS-SEM model were adapted. The first phase evaluated the study’s outer model for reliability and validity, while the second phase tested the inner model for hypothesis testing.

### 4.1. Phase One: Measurement Model

We assessed the measurement outer model validity and reliability, as per Hair et al.’s [33] recommendations, using measures such as “composite reliability” (CR) and “average variance extracted” (AVE) for each factor. Table 1 reports the model’s outer standardized factor loadings (FL), C.R., “Cronbach’s α”, and AVE score. The scale reliability indicated adequate and satisfactory results as all the reflective item loadings were above the suggested value of 0.50 [33]. Regarding CR values, all factors exceeded the lowest level of 0.70 score, as recommended by [42]. These results indicated that the study model met the reliability criteria. In terms of convergent validity, the AVE values presented in Table 1 exceed the recommended value of 0.50, as indicated by [43]. The final criterion of evaluation was to support DV [44] by inspecting the HTMT (“heterotrait–monotrait ratio of correlations”) values in Table 2. All values were below 0.90 [44], indicating adequate DV, and the Fornell-Larcker criterion, as depicted in Table 3, further supports DV, as all factors were found to load highly on their respective factors.

### 4.2. Phase-Two: Structural Inner Model Results

The structural model was assessed as recommended by [33] using several criteria, including “path coefficients, coefficient of determination (R^2^), predictive relevance (Q^2^), effect sizes (f^2^), multicollinearity diagnostics, and bootstrapping procedures”. Path significance was evaluated employing a bootstrapping process with 5000 resamples and “bias-corrected confidence intervals option”. “Variance Inflation Factor” (VIF) scores were inspected to evaluate multicollinearity, and all values were below the suggested threshold of 0.5, indicating the absence of multicollinearity [45]. Predictive relevance was evaluated using Stone–Geisser’s Q^2^ score, which exhibited acceptable predictive capability for the endogenous variables as suggested by [46] (Stress Q^2^ = 0.873, anxiety Q^2^ = 0.764, and depression Q^2^ = 0.549). Additionally, effect sizes (f^2^) were adequate for evaluating the contribution of each exogenous variable to the explained variance of the endogenous constructs. The structural model displayed strong explanatory power, with R^2^ values of 0.864 (Stress), 0.801 (anxiety), and 0.683 (depression). These results showed that the proposed model can explain a substantial proportion of the variance in university students’ MH consequences, supporting its adequacy and predictive power.

Regarding model goodness-of-fit (GoF) evaluation, the study recognises that PLS-SEM is primarily predictive-oriented rather than covariance-oriented. Thus, the regular GoF criteria widely employed in covariance-based SEM are not believed to be needed evaluation criteria in variance-based SEM methods. This proposition is confirmed by Henseler and Sarstedt [47], who stated that GoF metrics have limited diagnostic capacity in PLS-SEM. Nonetheless, the “Standardized Root Mean Square Residual” (SRMR) was additionally inspected (SRMS = 0.034) and was found to be below the cutoff point (0.08) as an approximate GoF criterion following the latest methodological recommendations [33].

**Figure 1:**
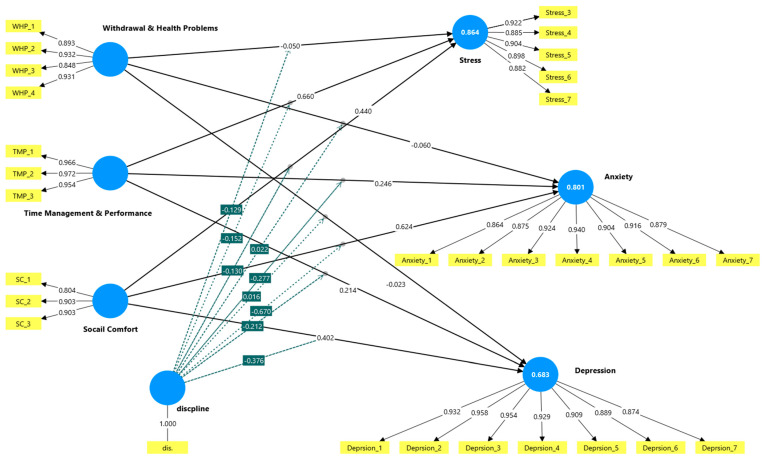
The PLS-SEM results revealed that withdrawal & health problems (W&HP) (as dimension of SMA) has negative significant associations with stress (β = −0.050, t = 3.201, *p* < 0.01) and anxiety (as mental health disorder) (β = −0.060, t = 1.967, *p* < 0.05), rejecting H1 and H3, and has negative insignificant relationship with depression, (β = −0.023, t = 0.657, *p* = 0.511) rejecting H2.

The outcomes signaled several statistically significant relationships between the latent dimensions, as shown in Figure 1 and Table 4.

**Figure 2:**
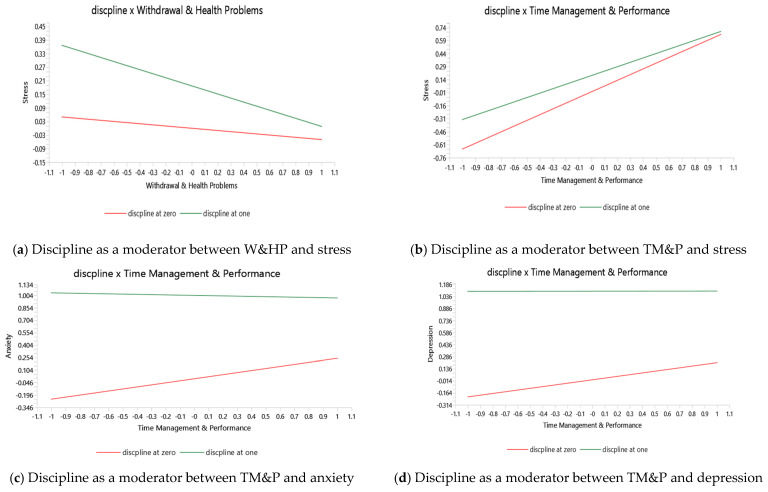

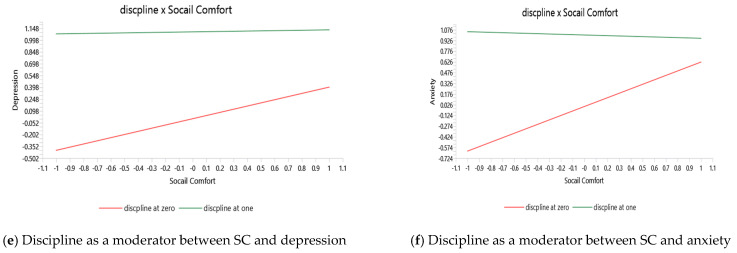
Simple slope for moderation effects.

Similarly, the report of PLS-SEM revealed that TM&P (as a dimension of SMA) has positive strong significant association with stress (β = 0.660, t = 13.891, *p* < 0.001); depression β = 0.214, t = 3.050, *p* < 0.01); and anxiety (β = 0.246, t = 4.849, *p* < 0.001), supporting H4, 5, and 6. Likewise, SC (as a dimension of SMA) was found to have positive significant association with stress (β = 0.440, t = 11.269, *p* < 0.001); depression (β = 0.402, t = 6.454, *p* < 0.001); and anxiety (β = 0.624, t = 14.765, *p* < 0.001), supporting H7, H8 and H9. Furthermore, the moderating effect of study discipline was examined to determine whether academic context can moderate the relationship between SMA dimensions and MHS. This analysis showed several significant differences across disciplinary fields. The results indicate that the moderating effect of study discipline is statistically significant (β = –0.129, *p* < 0.05), suggesting that the relationship between W&HP and Stress differs across study disciplines. Specifically, the slope for students in social science disciplines (green line) is steeper, indicating a stronger positive association between withdrawal symptoms and stress levels. In contrast, students in natural science disciplines (red line) exhibit a flatter slope, indicating a weaker relationship between these constructs, which supports H10. Likewise, the slope analysis supports the moderating hypotheses as shown in Figure 2 and Table 4, confirming that study discipline moderates the effect of SMA (TM&P) on stress, depression, and anxiety among SA university students, supporting H13, H14, and H15. Moreover, study discipline was found to significantly moderate the effect of SMA (SC) on depression and anxiety among SA university students, supporting H17 and H18.

## 5. Discussion

The results offered partial support for the suggested theoretical model, as several hypotheses were supported while others were rejected, emphasizing the complex and multidimensional nature of social media addiction (SMA) and its emotional outcomes. The PLS-SEM results revealed that W&HP had a negative and significant association with stress and anxiety, indicating that higher levels of W&HP were associated with lower symptoms of stress and anxiety, thereby rejecting H1 and H3 and contrasting with previous literature reporting strong positive links between problematic social media use and psychological distress. At first glance, this finding appears inconsistent with both the zero-order correlations and the established literature, which generally reports that higher levels of problematic social media use are associated with increased psychological distress. However, these path coefficients should be interpreted strictly as conditional effects within the multivariate PLS-SEM framework, rather than as direct or bivariate relationships. In other words, each coefficient represents the unique effect of a given construct on stress and anxiety after controlling for the shared variance among all other dimensions of social media addiction included in the model. Accordingly, this result does not imply that withdrawal or health-related problems are protective against stress or anxiety. Rather, it suggests that when the general severity of social media addiction is held constant, the residual variance uniquely associated with withdrawal-related symptoms may capture a different underlying process that is not directly aligned with general distress. Therefore, these coefficients should be interpreted with caution as model-dependent, conditional associations that are sensitive to specification decisions in the PLS-SEM framework.

Previous studies consistently reported that SMA is positively associated with anxiety and stress symptoms, and that withdrawal symptoms often intensify negative emotional states. Additionally, the relationship between W&HP and depression was weak and non-significant, thereby failing to support H2. These findings align partially with previous literature, which argues strong links between problematic SM attitude and anxiety and stress (e.g., the excessive reviews linking SM overuse to high levels of anxiety, stress, and other psychological issues) [2,48]. Previous studies consistently stated that SMA correlates positively with high levels of anxiety and stress disorder (e.g., [49]), and that withdrawal symptoms (e.g., discomfort when deprived of SM usage) often strengthen negative emotional states. However, the absence of a significant correlation with depression is somewhat divergent from many previous studies that showed robust correlations between SMA and depressive symptoms [50,51]). One reasonable explanation is that depressive symptoms frequently develop over longer time spans, necessitating more sustained exposure to risk dimensions. At the same time, stress and anxiety may be apparent in more acute responses to withdrawal and health-related issues.

The PLS-SEM report revealed that TM&P showed a strong positive and significant relationship with stress, depression, and anxiety, supporting H4, H5, and H6. These results revealed that university students who have some trouble in managing their time due to severe social media usage are more likely to show high levels of emotional distress. This is aligned with previous research and signaled that problematic social media usage is associated with worsened academic performance, delay, and higher levels of anxiety and stress [2,52]. Inadequate time management may also cause academic burnout, extra exacerbating the depression symptoms [53]. Similarly, SC, as another dimension of SMA was found to positively and significantly correlate with stress, depression, and anxiety, supporting H7, H8, and H9. This implies that students who are highly dependent on social media for emotional support and regular social relationships might be more subject to mental health concerns. While online social involvement can provide instant relief and a sense of connectedness, overdependence on online validation can lead to a higher level of social comparison, the fear of missing out (FoMO), and emotional stress [49,54]. Such consequences can mirror the “compensatory Internet use theory”, which argues that people might turn to online network sites to cope with adverse feelings and emotions, reinforcing addictive behavior and psychological strain [4].

This paper also found that study discipline can considerably moderate the association between SMA dimensions and mental health outcomes among SA university students. More specifically, the moderating effect of study discipline on the link between W&HP and stress (H10 was supported) revealed that study discipline meaningfully shapes how SMA can be translated into psychological issues. The slope analysis showed that university students in the social science field demonstrated a stronger positive association between W&HP signs and stress, whereas those in the natural science field reported a weaker association. This suggested that university students in fields with higher levels of social involvement (such as social sciences) might be more susceptible to emotional distress when disconnected from social platforms [49,55]. In contrast, students enrolled in natural science disciplines might be more instrumentally engaged with digital platforms, regularly for informational or academic purposes, and therefore are exposed to a lower level of withdrawal-related indicators [56]. The moderation association were further supported for the dimensions of TM&P and SC. The significant moderating core role of student discipline reported that the magnitude of the association between both TM&P mental health outcomes (stress, depression, and anxiety) and the association between SC and depression and anxiety (H17 and H18 were supported) differs across study disciplines (H13, H14, and H15 were supported). These outcomes are consistent with prior findings showing that academic capacity, study setting, and discipline-specific learning patterns can either intensify or buffer the psychological outcomes of social media use [57]. For example, university students in social science disciplines may be subjected to heightened levels of anxiety and stress due to a higher level of exposure to peer comparison and online social burdens. In contrast, those in natural science disciplines may exhibit better time management and a lower emotional vulnerability [54].

The results of this research offer some important practical implications for university leaders, mental health specialists, and policymakers in SA. The observed associations between the Time Management and Performance (TM&P) and Social Comfort (SC) dimensions of social media addiction and psychological distress symptoms suggest that universities should formulate targeted interventions to encourage healthy digital behaviour and balanced SM use among university students. Additionally, universities might benefit from implementing digital well-being platforms, time management programs, psychological support services, and awareness campaigns highlighting the risks associated with excessive social media use. Furthermore, the significant moderating effect of study discipline suggests that university students across different fields may experience varying levels of emotional vulnerability associated with social media use. Consequently, universities should recognise discipline-specific support procedures tailored to the academic demands and learning settings of different student disciplines. These results can also help policymakers and support centres propose evidence-based mental health programs that improve students’ emotional well-being in an increasingly digital educational setting.

## 6. Limitations and Upcoming Research Opportunities

Like previous studies in the social sciences, this study has some limitations. First, the cross-sectional approach adopted in this study might limit its ability to infer causality from the tested relationships. Consequently, the observed associations should be interpreted as correlational rather than causal, and future research would benefit from longitudinal or experimental designs to better establish temporal ordering and causality. Second, the data were collected using a convenience-based online sampling approach, which may introduce selection bias and limit the representativeness of the sample. As such, the generalizability of the results should be interpreted with caution, particularly beyond the context of Saudi university students from whom the data were drawn. Third, the exclusive reliance on self-report questionnaires may have introduced common method variance as well as social desirability bias, potentially inflating or biasing the observed relationships. Although procedural remedies were considered, the absence of behavioral or objective indicators of social media use further limits the robustness of the measurement approach. Future research should therefore incorporate multi-source data, digital trace data, or platform-based usage metrics alongside self-reports to enhance measurement validity and reduce common method bias. Finally, while the moderating role of study discipline yielded meaningful insights, this finding should be interpreted with caution. The discipline categories used in this study may be relatively broad and internally heterogeneous, which could mask important within-group differences. Moreover, the potential moderating effects should not be overgeneralized without further replication across more fine-grained academic classifications. Future studies are encouraged to examine additional moderating variables such as gender, academic level, and cultural context to provide a more nuanced and comprehensive understanding of how social media addiction relates to mental health outcomes.

## 7. Conclusions

This research offered good empirical evidence on the complicated associations between the three dimensions of SMA (W&HP, TM&P and SC) and MHS (stress, anxiety, and depression) among SA university students. Moreover, the study explored the key role of moderating study discipline in the tested relationships. PLS-SEM was used as the main data analysis method; the results showed that TM&P and social comfort dimensions of SMA can positively and significantly be associated with higher levels of stress, depression, and anxiety. On the contrary, W&HP reported a weaker, inconsistent association with mental health outcomes, suggesting that different SMA factors can be related to university students’ psychological well-being in unique contexts. The moderation analysis further showed that the link between SMA and mental health outcomes can be significantly associated with study discipline. Obviously, university students in the social sciences showed a stronger association between W&HP indicators and stress than those in other natural science disciplines. These results emphasise how disciplinary context is shaped by communication intensity, social burden, and study schedules, which can either intensify or buffer the psychological consequences of excessive social media use.

## Figures and Tables

**Table 1:** Psychometric properties of the employed scale.

Factors	Items	FL	*a*	CR	AVE	VIF
Anxiety			0.961	0.962	0.811	
	Anty_1	0.864				3.274
	Anty_2	0.875				3.650
	Anty_3	0.924				2.349
	Anty_4	0.940				1.437
	Anty_5	0.904				1.222
	Anty_6	0.916				1.827
	Anty_7	0.879				3.481
Depression			0.970	0.972	0.848	
	Dprson_1	0.871				1.397
	Dprson_2	0.900				2.724
	Dprson_3	0.874				4.208
	Dprson_4	0.880				2.311
	Dprson_5	0.910				3.641
	Dprson_6	0.902				3.562
	Dprson_7	0.904				3.397
Social comfort			0.841	0.843	0.759	
	SC_1	0.804				1.381
	SC_2	0.903				3.067
	SC_3	0.903				3.054
Stress			0.958	0.959	0.797	
	Strs_1	0.870				3.527
	Strs_2	0.922				1.494
	Strs_3	0.886				1.916
	Strs_4	0.885				1.905
	Strs_5	0.904				2.332
	Strs_6	0.898				1.103
	Strs_7	0.882				3.885
Time management & Performance			0.962	0.962	0.930	
	TMP_1	0.966				3.153
	TMP_2	0.972				3.186
	TMP_3	0.954				3.303
Withdrawal and health problems			0.923	0.927	0.813	
	WHP_1	0.893				2.909
	WHP_2	0.932				3.483
	WHP_3	0.848				2.425
	WHP_4	0.931				3.422

**Table 2:** Heterotrait–monotrait ratio (HTMT)—Matrix.

	1	2	3	4	5	6
1-Anxiety						
2-Depression	0.778					
3-SC	0.742	0.760				
4-Stress	0.735	0.647				
5-TM&P	0.617	0.573	0.793	0.781		
6-W&HP	0.426	0.436	0.411	0.458	0.584	

**Table 3:** Fornell–Larcker Table.

	1	2	3	4	5	6
1-Anxiety	0.901					
2-Depression	0.848	0.921				
3-SC	0.779	0.706	0.871			
4-Stress	0.733	0.623	0.838	0.893		
5-TM&P	0.593	0.554	0.718	0.864	0.964	
6-W&HP	0.403	0.414	0.456	0.390	0.434	0.902

**Table 4:** Results of the Structural Model.

	[β]	[*T*]	[*p*]	Conclusions
W&HP -> Stress	−0.050	3.201	0.001	H1-Rejected
W&HP -> Depression	−0.023	0.657	0.511	H2-Rejected
W&HP -> Anxiety	−0.060	1.967	0.049	H3-Rejected
TM&P -> Stress	0.660	13.891	0.000	H4-Supported
TM&P -> Depression	0.214	3.050	0.002	H5-Supported
TM&P -> Anxiety	0.246	4.849	0.000	H6-Supported
SC -> Stress	0.440	11.269	0.000	H7-Supported
SC -> Depression	0.402	6.454	0.000	H8-Supported
SC -> Anxiety	0.624	14.765	0.000	H9-Supported
Moderation effects				
Discipline × W&HP -> Stress	−0.129	2.477	0.013	H10-Supported
Discipline × W&HP -> Depression	0.016	0.224	0.823	H11-Rejected
Discipline × W&HP -> Anxiety	0.022	0.399	0.690	H12-Rejected
Discipline × TM&P -> Stress	−0.152	2.271	0.023	H13-Supported
Discipline × TM&P -> Depression	−0.212	2.513	0.012	H14-Supported
Discipline × TM&P -> Anxiety	−0.277	4.787	0.000	H15-Supported
Discipline × SC -> Stress	−0.130	1.646	0.100	H16-Rejected
Discipline × SC -> Depression	−0.376	4.093	0.001	H17-Supported
Discipline × SC -> Anxiety	−0.670	11.205	0.000	H18-Supported

## Data Availability

The data presented in this study are available on request from the corresponding authors due to privacy and ethical restrictions.
